# Lipid profile status and other related factors in patients with Hyperphenylalaninaemia

**DOI:** 10.1186/s13023-016-0508-x

**Published:** 2016-09-09

**Authors:** María L. Couce, Isidro Vitoria, Luís Aldámiz-Echevarría, Ana Fernández-Marmiesse, Iria Roca, Marta Llarena, Paula Sánchez-Pintos, Rosaura Leis, Alvaro Hermida

**Affiliations:** 1 Unit of Diagnosis and Treatment of Congenital Metabolic Diseases. S. Neonatology, Department of Pediatrics, Hospital Clínico Universitario de Santiago, CIBERER, Health Research Institute of Santiago de Compostela (IDIS), Travesía da Choupana s/n, 15706 Santiago de Compostela, A Coruña Spain; 2 Unit of Metabolopathies, Hospital Universitario La Fe, Bulevar sur s/n, 46021 Valencia, Spain; 3 Unit of Metabolism. Cruces University Hospital, Biocruces Health Research Institute, GCV-CIBER de Enfermedades Raras (CIBERER), Plaza de Cruces s/n, 48903 Barakaldo, Vizcaya Spain; 4 Unit of Gastroenterology and Nutrition, Department of Pediatrics, Hospital Clinico Universitario de Santiago, Health Research Institute of Santiago de Compostela (IDIS), Travesía da Choupana s/n, 15706 Santiago de Compostela, A Coruña Spain; 5 Unit of Diagnosis and Treatment of Congenital Metabolic Diseases, Department of Internal Medicine, Universidad de Santiago, Santiago de Compostela, Spain

**Keywords:** Atherogenic profile, Blood pressure, Coronary heart disease, Homocysteine, Lipoprotein, Phenylketonuric dietary treatment

## Abstract

**Background:**

The mainstay of treating patients with phenylketonuria (PKU) is based on a Phe-restricted diet, restrictive in natural protein combined with Phe-free L-amino acid supplements and low protein foods. This PKU diet seems to reduce atherogenesis and confer protection against cardiovascular diseases but the results from the few published studies have been inconclusive. The aim of our study was to evaluate the relationship between the lipid profile and several treatment-related risk factors in patients with hyperphenylalaninaemia (HPA) in order to optimize their monitoring.

**Methods:**

We conducted a cross-sectional multicentre study. A total of 141 patients with HPA were classified according to age, phenotype, type of treatment and dietary adherence. Annual median blood phenylalanine (Phe) levels, Phe tolerance, anthropometric measurements, blood pressure (BP) and biochemical parameters [(triglycerides, total cholesterol (TC), high-density lipoprotein cholesterol (HDL-C), low density lipoprotein-cholesterol (LDL-C), apolipoprotein A (ApoA), apolipoprotein B (ApoB), vitamin B_12_, total homocysteine (tHcy), Methionine (Met), high sensitivity C-Reactive Protein (hsCRP)] were collected for each patient.

**Results:**

Plasma TC levels were lower in patients with PKU than in the mild-HPA group (150 ± 31 vs. 164 ± 22 mg/dL), and there was a weak inverse correlation between plasma TC and Phe levels. HDL-C, LDL-C, ApoA and ApoB levels were lower in the PKU group than in mild-HPA. Patients with PKU had higher systolic BP than the mild-HPA group and there was found a quadratic correlation between median Phe levels and systolic BP (*p* = 6.42e^-5^) and a linear correlation between median Phe levels and diastolic BP (*p* = 5.65e^-4^). In overweight or obese PKU patients (24.11 %), biochemical parameters such as TC, triglycerides, LDL-C, tHcy, hsCRP and BP were higher. By contrast, HDL-C was lower in these patients.

**Conclusion:**

Our data show a direct correlation between lipid profile parameters and good adherence to the diet in PKU patients. However, lipid profile in overweight or obese patients displayed an atherogenic profile, in addition to higher hsCRP concentrations and BP. Our study contributes to a better understanding of the relationship between phenotype and treatment in patients with HPA, which could be useful in improving follow-up strategies and clinical outcome.

**Trial registration:**

Research Ethics Committee of Santiago-Lugo 2015/393. Registered 22 September 2015, retrospectively registered.

**Electronic supplementary material:**

The online version of this article (doi:10.1186/s13023-016-0508-x) contains supplementary material, which is available to authorized users.

## Background

Phenylketonuria (PKU; MIM 261600), an autosomal recessive disorder of Phenylalanine (Phe) metabolism, is mainly caused (98 % of cases) by deficient activity of the hepatic enzyme L-phenylalanine-4-hydroxylase (PAH; EC 1.14.16.1), due to mutations in the *PAH* gene (NM 000277.1). Loss of PAH activity results in increased blood Phe concentrations which leads to irreversible neurological damage if it is untreated. Average incidence in Caucasians is of 1 in 10,000 individuals [1]. Hyperphenylalaninaemia (HPA) is usually diagnosed by Newborn Screening Programme, routinely performed in Spain since 1980 [2], which enable early diagnosis and treatment. Pre-established thresholds for plasma Phe concentrations according to age are not yet standardized and vary among countries [3, 4].

Despite the appearance of novel approved treatment approaches (for example, large neutral amino acids, (6R)-L-erythro-5,6,7,8-tetrahydrobiopterin (6R-BH_4_) and glycomacropeptide), dietary intervention remains the mainstay of treating patients with PKU [5]. Patients are typically recommended to follow a PKU diet based on low Phe intake. This diet consists of a marked reduction in natural protein, with supplementation with Phe-free amino acid mixture.

There is a general agreement that patients with PKU require long-term dietary counselling as well as daily nutritional supplementation [5]. This diet has some drawbacks, such as the poor organoleptic properties (taste, scent) of the Phe-free protein substitutes and special-manufactured low-protein foods which are likely to have a negative effect on compliance with the diet. Furthermore, long-term safety of this dietary treatment as well as its potential association with risk of non-communicable diseases in later stages should be further investigated [6]. In this regard, there are several recently published reports about the contribution of mineral and vitamin status [7–11]. However, very few studies have explored the blood lipid profile in PKU [12, 13]. In particular, it has been observed that PKU children who had good adherence to diet, consumed saturated (below 7 %) and polyunsaturated (above 5 %) fats from total daily energy with amounts below 50 mg of cholesterol per day [14].

The aim of our study was to determine the blood lipid profile status and several cardiovascular-related risk factors, such as overweight/obesity, plasma total homocysteine (tHcy) levels, high-sensitivity C-reactive protein (hsCRP), micronutrients (zinc (Zn) and cupper (Cu)) and blood pressure (BP) in patients with PKU, who were classified into groups according to Phe tolerance, 6R-BH_4_ supplementation and adherence to diet. Moreover, we further analysed potential risk factors due to its deficiency or over supplementation.

## Methods

### Study design

A total of 141 patients were enrolled in this cross-sectional multicentre study between February 2015 and February 2016 from three Metabolic Diseases Unit at three Spanish Hospitals: Hospital Universitario La Fe (Valencia) (*n* = 62), Hospital Universitario Cruces (Basque Country) (*n* = 23) and Hospital Clínico Universitario de Santiago (Galicia) (*n* = 56). The study protocol was approved by the Research Ethics Committee of Santiago-Lugo (2015/393). Written informed consent was obtained from parents or legal guardians of children (below 16 years of age) and patients included. Patients were followed in their respective Centre from the date of diagnosis up to current date. The study included both patients who were diagnosed through Newborn Screening Programmes as well as patients who were later diagnosed due to the appearance of clinical symptoms. Exclusion criteria were: (1) poor medical monitoring, changes in amino acid mixture during the month prior to enrolment and (3) pregnancy.

Data collected for each patient were: age, gender, phenotype [patients were classified into phenotypic categories according to blood Phe levels, which were measured at diagnosis, based on US Guidelines [15]: mild-hyperphenylalaninaemia (MHPA), (120–360 μmol/L); mild-moderate PKU (MPKU), (360–1200 μmol/L) and classic PKU (CPKU), (>1200 μmol/L)], diagnosis time (early versus late diagnosis), annual median blood Phe levels (pre-established “safe” thresholds: <360 μmol/L for children below 6 years of age; <480 μmol/L for those from 6 to ≤10 years of age and ≤600 μmol/L for those >10 years of age) [2], anthropometric characteristics [weight, height, body mass index (BMI), waist circumference (WC) and mid-upper arm circumference (AC)], Phe tolerance (low <500 mg/day, high >500 mg/day), 6R-BH_4_ therapy (treated vs. non-treated), systolic and diastolic blood pressure (SBP and DBP), and blood biochemical measurements such as: triglycerides (TGC), total cholesterol (TC), high-density lipoprotein cholesterol (HDL-C), low density lipoprotein-cholesterol (LDL-C), apolipoprotein A (ApoA), apolipoprotein B (ApoB), Zn, Cu, vitamin B_12_ (B_12_), total homocysteine (tHcy), Methionine (Met). LDL cholesterol/ApoB and Zn/Cu ratios were calculated. High-sensitivity C-Reactive Protein (hsCRP) was measured in 55 patients. Blood samples for the measurements were obtained after overnight fasting at the same time (8:00 h in the morning) from patients without acute infection or medication (except from patients on 6R-BH_4_ therapy). Reference range values (valid for all ages) for micronutrients were: vitamin B_12_ (180-1900 pg/mL); Zn (65- 140 μg/dL); Cu (65-140 μg/dL); tHcy (5-15 μmol/L); hsCRP (0-1.1 mg/dL) and Met (10-60 μmol/L for patients < 18 years of age; 20-37 μmol/L for patients > 18 years of age). With regard to cardiovascular parameters the upper thresholds were: TC > 200 mg/dL, LDL-C > 130 mg/dL; ApoB ≥ 110 mg/dL and TGC > 100 mg/dL for patients from 0 to 9 years of age and >130 mg/dL for patients >10 years of age. Lower thresholds were: TGC < 30 mg/dL; HDL-C < 40 mg/dL and ApoA < 115 mg/dL. Adherence to treatment in patients with PKU was established according to their metabolic control by annual median blood Phe levels and the pre-established “safe” thresholds for each age (as above mentioned) [2]. Definition and staging of high blood pressure (BP) in children and adolescents were based on BP at presentation according to US National High Blood Pressure Education Program [16]. High blood pressure was defined as systolic BP (SBP) and/or diastolic BP (DBP) above the 95th percentile according to gender, age, and height; while prehypertension was defined as SBP and/or DBP from the 90th to 95th percentile on repeated measurements. Diagnosis of essential hypertension in adults was established according to definitions provided by the European Society of Hypertension and the European Society of Cardiology guidelines [17].

### Methods

The three Centres followed the same protocol. Dietary treatment was based on recommendations in the Spanish Guidelines for treating and monitoring patients with PKU [2]. That is, patients are typically recommended to follow a PKU diet, which consists of a marked reduction in natural protein diet, and supplementation with Phe-free amino acids mixture. Average protein intake was 1.3-1.5 times above Recommended Dietary Allowances (RDA) [18]. Nutritional diet was weekly assessed by 3-day food surveys completed using software www.odimet.es. The group of MHPA patients, who had Phe levels at diagnosis of 120-360 μM/L, did not require treatment and therefore they followed a normal diet.

Recumbent length was measured with a measuring board and weight with a manual baby scale until the age of 24 months. Thereafter, standing height was measured with a wall-mounted stadiometer and body weight, with digital scales. Patients were weighed barefoot and after overnight fasting. The nutritional status was assessed by calculating the body mass index (BMI) using the formula BMI = weight (kg) /height^2^ (m^2^). Patients above 18 years of age were classified based on World Health Organization (WHO) criteria as: underweight (BMI < 18.5), normal weight (BMI 18.5-24.99), overweight (BMI 25-29.99), and obese (BMI ≥ 30). Patients below 18 years of age were classified according to BMI using the WHO Child Growth Standards (Underweight: BMI below the 15th percentile; Normal: BMI from the 15th to 85th percentiles, Overweight: BMI from the 85th to 95th percentiles, Obese: BMI above the 95th percentile) [19, 20].

Waist circumference (WC) was measured at a level midway between the lower rib margin and the iliac crest and mid-upper arm circumference (AC) was measured at a point half-way between the elbow and the shoulder. These measurements (expressed in cm) were stratified according to gender and age based on the previously published study in children named Estudio Galinut [21]. In adult patients, specific values for WC and AC were used from the International Diabetes Federation (IDF) [22] and Frisancho’s study [23], respectively.

Phe levels from dried blood spots and plasma samples were measured using Tandem mass-spectrometry. TC, HDL-C and TGC concentrations were determined by standard procedures using an Advia 2400 Analyzer (Siemens Diagnostic Systems, Germany). When TGC levels were < 350 mg/dL, LDL-C was estimated using the Friedewald formula LDL = TC - HDL-C - TG/5.0 (mg/dL). When TGC levels were >350 mg/dL, LDL-C was estimated by a direct method which was based on cholesterol oxidase, esterase and peroxidase, once lipoprotein cholesterol (other than LDL) were removed using an Advia 2400 Analyzer (Siemens Diagnostic Systems, Germany). Vitamin B_12_ was determined using an Advia Centaur XP Analyzer (Siemens Healthcare Diagnostics, Erlangen, Germany), Zn and Cu using a 7700 ICP-Mass Spectrometry System (Agilent, CA, USA). Plasma ApoA and ApoB levels were measured by immunonephelometry assay using a Dimension Vista™ 1500 Analyzer (Siemens Healthcare Diagnostics) and tHcy concentrations were determined in EDTA-plasma samples by a competitive immunoassay using an IMMULITE® 2000 Analyzer (Siemens Healthcare Diagnostics). hsCRP levels were determined by spectrophotometry.

BP was measured using an oscillometric device (Omron IT-750; Omron Healthcare, Tokyo, Japan) and an appropriate cuff was applied in the supine position after a resting period of 5 min. Patients were asked not to practice any physical activity and to avoid caffeinated beverages prior to medical appointments. Simultaneous supine measurements at both arms were recorded, and shortly afterward three additional supine measurements were taken at the arm with displayed the higher BP. Average of the last two measurements was used for data analysis.

### Statistical test

In order to determine significant associations and/or differences between the different variables measured, the following methods were applied: first of all, we used the Kolmogorov–Smirnov and the Shapiro–Wilk tests to determine if the groups follow a normal distribution. Later, if one of the variables was quantitative and the other qualitative, we applied the Student *t*-test or the ANOVA if the quantitative variable was normal, and the Wilcoxon signed-rank or the Kruskal–Wallis test otherwise. If both variables were quantitative, under normality assumptions we fitted a linear regression model, and the p-value is the significance level of the adjustment; if the data wasn’t normal, we used the Kruskal–Wallis test. In the case that both variables were qualitative we used Fisher’s exact test. Finally, we adjusted the obtained p-values using the Benjamini-Hochberg correction. Only adjusted p-values lower than 0.05 were considered significant. Statistical analysis was performed using R Core Team [24], version 3.2.3.

## Results

Our study included 141 patients with HPA [67 (47.5 %) males; range age: 6 months-50 years; mean age: 15 years 6 months]. There were 100 patients (71 % of our population) who were diagnosed with PKU and 41 (29 %) with MHPA**.** In the group of patients with PKU, 66 (66 %) were classified as CPKU and 34 (34 %) as MPKU. A total of 16 (11.3 %) of our patients were diagnosed at a later stage by the appearance of clinical symptoms, due to the fact that in Spain the Newborn Screening Program for PKU started in the 70-80’s. With regard to 6R-BH_4_ therapy, 17 (17 %) of the patients with PKU were treated, out of them 14 (82.3 %) were classified as MPKU.

Over the observation period, adequate dietary adherence was observed in 120 patients (85.1 % of our population); in the group of patients below 10 years of age (63), 56 (88.9 %) had adequate dietary adherence; whereas in the group of patients above 10 years of age (78), 14 (18 %) had low dietary adherence,13 (92.8 %) of these patients were above 18 years of age [5 (62.5 %) of them where diagnosed at a later stage]. Only 1 (5.9 %) out of the 17 patients who were on 6R-BH_4_ treatment displayed Phe levels slightly above the pre-established “safe” threshold for their age ((P.23) Table 1 and Additional file 1).

**Table 1:** Clinical and biochemical parameters for patients with MHPA and PKU; *BMI* body mass index, *WC* waist circumference, *AC* arm circumference, *BP* blood pressure

	MHPA	PKU
Number patients	41	100
Gender – Male	20	47
Gender – Female	21	53
Average Age	9y 7 m	17y 10 m
Under 18 years	35	57
zBMI – obese + overweight	3	34
zBMI – normal	33	61
zBMI – underweight	5	5
WC - obese + overweight	7	45
WC - normal	30	50
WC - underweight	4	5
AC - obese + overweight	5	21
AC - normal	21	27
AC - underweight	4	15
Early Diagnostic	40	85
BH4 treatment	0	17
Adequated Diet adherence	40	80
Phe Tol (mg/day)	1889.6 ± 831.9	481.1 ± 376.3
Phe median (μmol/L)	238.6 ± 93.2	397.3 ± 281.9
Total Cholesterol (mg/dL)	164.2 ± 21.9	149.9 ± 31.5
HDL (mg/dL)	55.8 ± 12.6	49.8 ± 11.9
LDL (mg/dL)	92.1 ± 17.2	82.1 ± 24.7
ApoA (mg/dL)	160.7 ± 26.4	144.1 ± 25.9
ApoB (mg/dL)	75.5 ± 17.6	65.9 ± 19.1
LDL/ApoB	1.26 ± 0.16	1.22 ± 0.19
Triglycerids (mg/dL)	79.2 ± 36.2	91.7 ± 45.7
Homocystein (μmol/L)	5.9 ± 2.4	5.9 ± 3.3
Systolic BP (mmHg)	105.5 ± 12	114.9 ± 14.4
Diastolic BP (mmHg)	65.7 ± 10.5	69.3 ± 13.7
B12 (pg/mL)	584.9 ± 205.6	664.5 ± 333.6
Zinc (μg/dL)	82.4 ± 33.1	81.2 ± 21.5
Copper (μg/dL)	86 ± 22.5	78.4 ± 21.3
Zinc/Copper	1.01 ± 0.46	1.11 ± 0.45

**Fig. 1:**
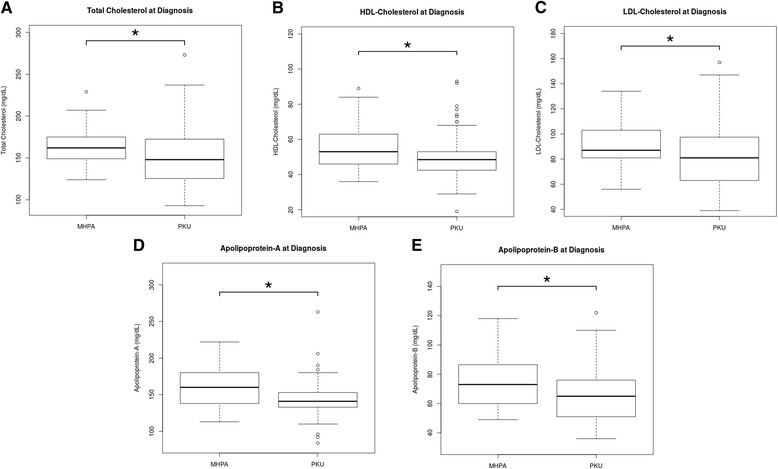
Lipid profile in patients with phenylketonuria vs. patients with mild hyperphenylalaninaemia. Box-plot diagrams showing the levels measured at diagnosis: **a**) Total Cholesterol (mg/dL), **b**) high-density lipoprotein (HDL)-Cholesterol (mg/dL), **c**) low density lipoprotein (LDL)-Cholesterol (mg/dL), **d**) Apolipoprotein-A (mg/dL) and **e**) Apolipoprotein-B (mg/dL). Box-plots show median values (solid horizontal line); 25th and 75th percentiles (box); minimum and maximum values (bars); outliers (open circles). The * indicates the statistical significant differences: * *p* < 0.05, using the statistical tests described in the Methods Section. PKU: phenylketonuria; MHPA: mild hyperphenylalaninaemia

With regard to blood lipid profile, TGC deficiency was observed in only 2 patients (1.4 %), but it was at the upper threshold in 26 patients (18.4 %), none of them was on 6R-BH_4_ treatment. Nevertheless, TC was decreased in 19 patients (13.5 %) (100 % of them were patients with PKU), out of them 14 (73.7 %) were below 18 years of age and 16 (84.2 %) of them had an adequate adherence to treatment. Similarly, HDL-C, LDL-C, ApoA and ApoB concentrations were below the low threshold in the PKU group compared with the MHPA group (Table 2, Fig. 1). However, in the group of PKU patients, those who had a good metabolic control displayed higher HDL-C concentrations and LDL/ApoB ratio than those with poor metabolic control (50.4 ± 12.02 vs. 47.4 ± 11.26 mg/dL and 1.22 ± 0.19 vs. 1.20 ± 0.23, respectively); although these differences did not reach statistical significance.

**Table 2:** Number of patients with hyperphenylalaninaemia with altered biochemical concentrations of lipid profile and other related parameters

	TC (<120 mg/dL)	TC (>200 mg/dL)	TGC(>100 mg/dL below 10 years of age >130 mg/dL above 10 years of age)	TGC (<30 mg/dL)	HDL-C (<40 mg/dL)	LDL-C (>130 mg/dL)	ApoA (<115 mg/dL)	ApoB (>110 mg/dL)	tHcy. (<5 μmol/L)	tHcy. (>15 μmol/L)	Zn (<65 μg/dL)	Cu (<65 μg/dL)	Cu (>140 μg/dL)	B_12_ (>1900 pg/mL)	hsCRP (>1.1 mg/dL)
over total of patients N (%)	19 (13.47)	6 (4.25)	26(18.44)	2(1.42)	17 (12.06)	4 (2.84)	6(4.25)	4(2.84)	58(41.13)	2(1.42)	28(19.86)	31(21.98)	3(2.13)	2(1.42)	5(3.55)
Age (below 18 years of age) N (%)	14 (73.68)	4 (66.67)	10(38.46)	1(50)	9(52.94)	1(25)	6(100)	2(50)	50(86.2) *p* = 3.18e^−4^	0(0)	23(82.14)	17(54.84)	0(0)	0(0)	0(0)
Gender (Male) N (%)	12(63.16)	3(50)	9(34.62)	0(0)	10(58.82)	1(25)	4(66.67)	2(50)	26(44.83)	2(100)	10(35.71)	12(38.7)	0(0)	0(0)	1(20)
PKU patients N (%)	19 (100) *p* = 0.0125	2(33.33)	21(80.77)	2(100)	14(82.35)	3(75)	5(83.33)	2(50)	42(72.41)	2(100)	19(67.86)	26(83.87)	1(33.33)	2(100)	5(100)
6R-BH_4_ therapy N (%)	1 (5.26)	0(0)	0(0)	1(50)	0(0)	0(0)	1(16.67)	0(0)	9(15.52)	0(0)	3(10.71)	4(12.9)	0(0)	0(0)	2(40)
Adherence to treatment (adequate) N (%)	16 (84.21)	6(100)	22(84.62)	1(50)	14(82.35)	3(75)	5(83.33)	4(100)	50 (86.2)	1(50)	26(92.86)	26(83.87)	3(100)	2(100)	4(80)
Phe tolerance (under 500 mg/day) N (%)	18 (94.73) *p* = 2.29e^−4^	3(50)	22(84.62)	2(100)	13(76.47)	2(50)	5(83.33)	3(75)	24(41.38)	0(0)	8(28.57)	22(70.97)	1(33.33)	0(0)	3(60)

*p-values were obtained using Fisher’s exact test and adjusted by Benjamini-Hochberg’s correction to determine statistically significant differences between patients with deficient micronutrient concentrations andpatients in the normal range according to: age (below/above 18 years of age), gender (male/female), diagnosis (PKU / MHPA), 6R-BH_4_ therapy (yes/no), adherence to dietary treatment (adequate/inadequate) and Phe tolerance (below/above 500 mg/day). Statistically significant differences are shown in the Table (*p* < 0.05). *N* number of patients, *%* percentage of patients, *TC* total cholesterol, *TGC* triglycerides, *HDL-C* high-density lipoprotein cholesterol, *LDL-C* low- density lipoprotein cholesterol, *ApoA* apolipoprotein A, *ApoB* apolipoprotein B, *tHcys* total homocysteine, *Zn* Zinc, *Cu* Cupper, *B*_*12*_ vitamin B_12_, *hsCRP* high sensitivity C-Reactive Protein, *PKU* phenylketonuria, *MHPA* mild hyperphenylalaninaemia, *Phe* phenylalanine

B_12_ levels were in the normal range or even slightly elevated in all patients; Cu and Zn levels were decreased in 31 (22 %) and 28 (19.9 %) patients, respectively. In the group of patients with low Cu levels, 18 (58.1 %) were CPKU and in the group with low Zn levels, 13 (46.4 %) were CPKU; all of them received Phe free formula. Plasma tHcy levels were elevated only in two PKU patients, both of them displayed B_12_ levels in the normal range (362 pg/mL and 237 pg/mL, respectively), whereas its levels were decreased in 58 patients (41.1 %), mainly in patients with an adequate adherence to treatment (50 patients, 86.2 %) and with lower Phe tolerance (44 patients, 75.9 %). hsCRP was elevated in 4 patients (2.84 %), all of them were PKU patients who were on dietary treatment, above 18 years of age and with overweight/obesity. Met levels were in the normal range for all the patients (26.96 ± 4.2 μmol/L).

**Fig. 2:**
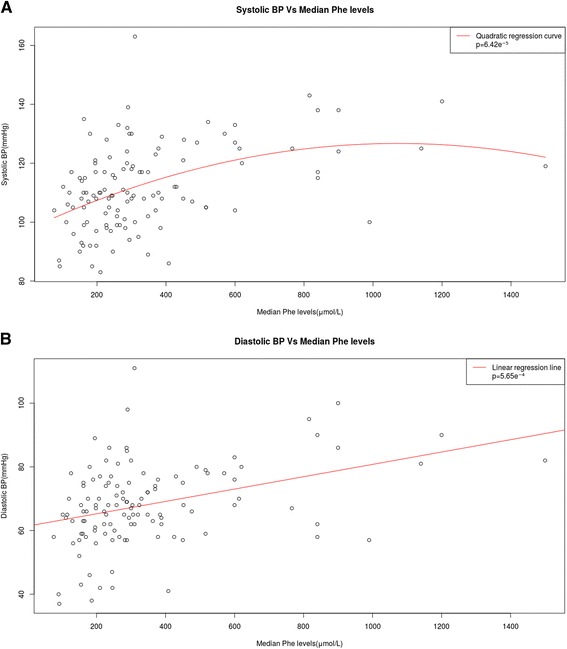
Correlations between systolic and diastolic blood pressures and mean phenylalanine levels in patients with phenylketonuria and mild hyperphenylalaninaemia. **a** Quadratic correlation between systolic blood pressure and median Phe levels. Statistical significance was observed (*p* < 0.001). **b** Linear correlation between diastolic blood pressure and median Phe levels. Statistical significance was observed (*p* < 0.001). Phe: phenylalanine (μmol/L); BP: blood pressure (mmHg)

Children and adults patients with PKU had higher systolic blood pressure (114.9 ± 14.4 vs. 105.5 ± 12.03 mmHg; *p* = 0.0438) than patients with MHPA. Although diastolic blood pressure was also higher in the PKU group compared with the MHPA group (69.3 ± 13.65 vs. 65.7 ± 10.49 mmHg), this difference did not reach statistical significance. It is worth mentioning that a statistically significant quadratic correlation was observed between median Phe and systolic BP (*p* = 6.42e^-5^), and a linear correlation between median Phe and diastolic BP (*p* = 5.65e^-4^), both in patients below and above 18 years of age (Fig. 2).

**Fig. 3:**
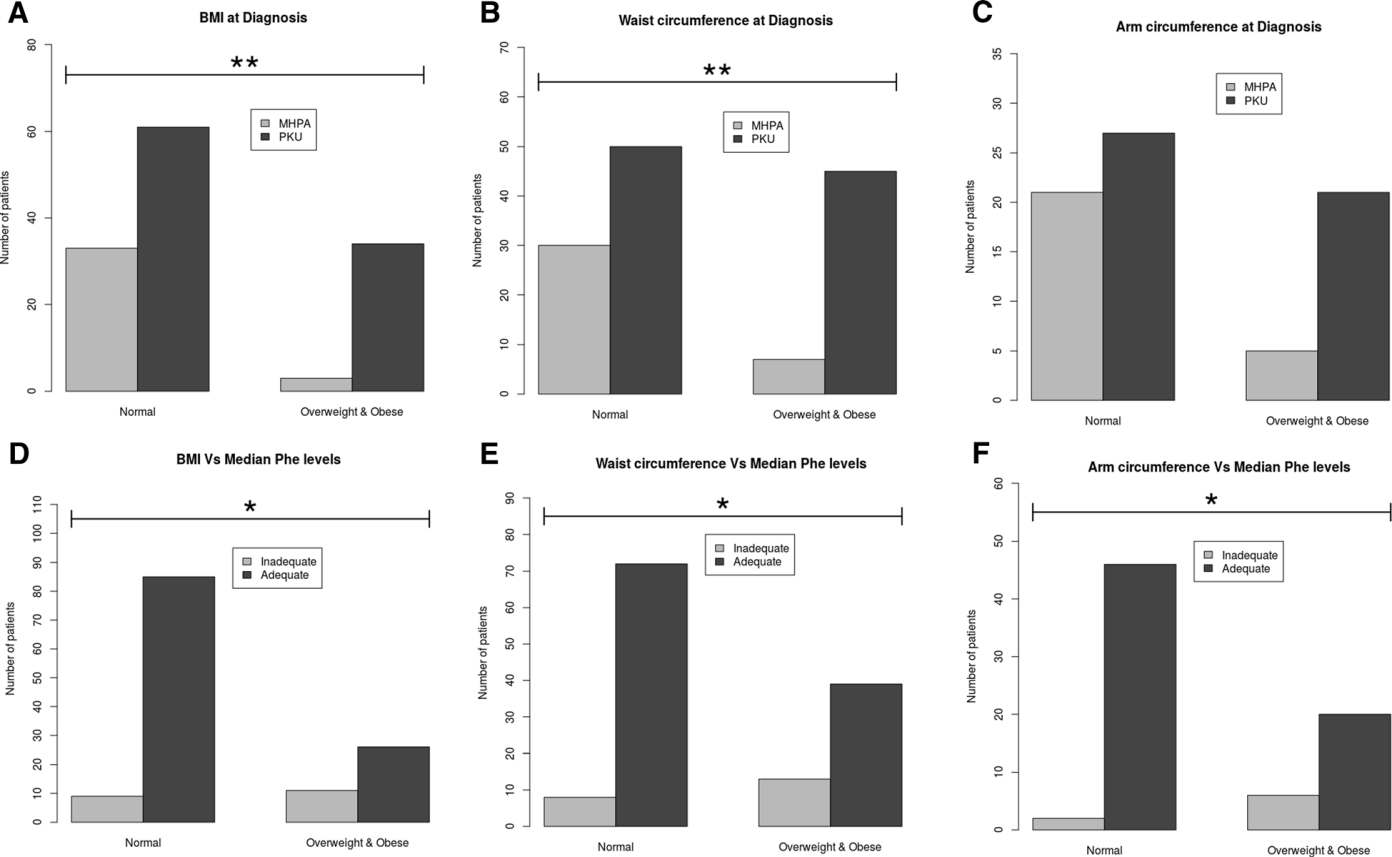
Body mass index, waist and arm circumference at diagnosis and in relation with median Phe levels in mild hyperphenylalaninaemia and phenylketonuria. The X-axis indicates the comparison between normal and overweight-obese groups in our population. The Y-axis indicates the number of patients. **a** Body mass index Z-score, **b**) Waist circumference and **c**) Arm circumference were measured at diagnosis in patients with mild hyperphenylalaninaemia () and phenylketonuria (). **d** Body mass index Z-score, **e**) Waist circumference and **f**) Arm circumference in relation with median Phe levels (μmol/L) in patients with poor () and good () metabolic control. Statistical significance was observed: * p < 0.05 and ** *p* < 0.01, using the Fisher’s exact test and the Benjamini-Hochberg correction. PKU: phenylketonuria; MHPA: mild hyperphenylalaninaemia; BMI: body max index; Phe: phenylalanine

With regard to body mass index (BMI), 47 patients (33.33 %) did not have an adequate BMI Z-score, which had a value below the lower threshold in 10 patients (7.1 %) and above the upper threshold in 37 patients (26.24 %) [25 (67.6 %) were overweight and 12 (32.4 %) were obese]. Waist circumference (WC) was above the recommended threshold in 52 (36.9 %) patients [27 (51.9 %) of them above the 95th percentile). Arm circumference (AC) was elevated in 26 (18.4 %) patients [11 (42.3 %) of them above the 95th percentile). The percentage of patients with elevated BMI Z-score and WC was significantly higher in the group of PKU patients compared with the MHPA group [34 (91.9 %) vs. 3 (8.1 %), *p* = 0.005 for BMI Z-score; and 45 (86.5 %) vs. 7 (13.5 %), *p* = 0.007 for WC]. When age of patients was considered, we observed that patients who had an elevated BMI Z-score were all above 18 years of age [37 (100 %) vs. 0 (0 %), *p* = 4.57e^-23^), and that a higher percentage of patients above 18 years of age had WC above the recommended thresholds [34 (65.4 %) vs. 18 (34.6 %), *p* = 5.63e^-7^], with only 2 (6.25 %) patients diagnosed with PKU. Furthermore, BMI Z-score, AC and WC were also statistically significantly higher in patients with good metabolic control compared to patients with poor metabolic control: 26 (70.3 %) vs. 11 (29.7 %) (*p* = 0.011) for BMI Z-score; 39 (76.9 %) vs. 13 (23.1 %) (*p* = 0.048) for AC; and 19 (75 %) vs. 4 (25 %) (*p* = 0.035) for WC (Fig. 3).

**Fig. 4:**
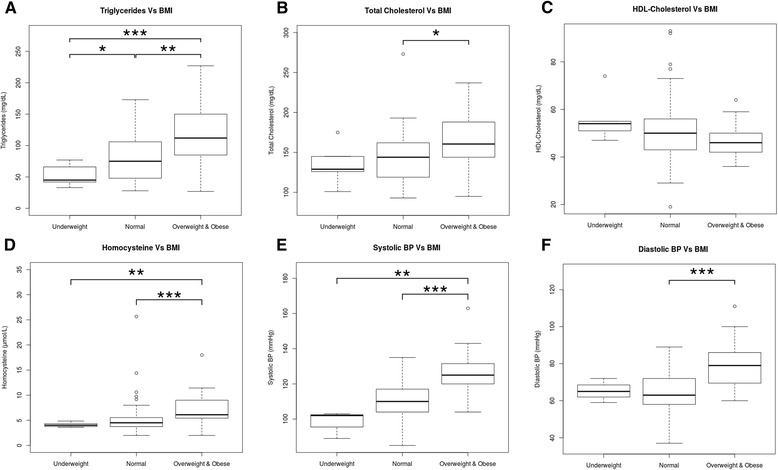
Biochemical parameters of lipid profile, homocysteine and blood pressure in patients with phenylketonuria classified according to their body mass index. Box-plot diagrams showing A) Triglycerides (mg/dL), B) Total Cholesterol (mg/dL), C) HDL-Cholesterol (mg/dL), D) Homocysteine (μmol/L), E) Systolic BP (mmHg) and F) Diastolic BP (mmHg). The X-axis indicates the underweight, normal and overweight & obese groups in our patients with phenylketonuria. Box-plots show median values (solid horizontal line); 25th and 75th percentiles (box); minimum and maximum values (bars); outliers (open circles). The * indicates the statistical significant differences: * p < 0.05, ** *p <* 0.01 and *** *p <* 0.001, using the statistical tests described in the Methods Section. BMI: body max index; HDL-cholesterol: high-density lipoprotein cholesterol; BP: blood pressure

Biochemical parameters such as TC, TGC, LDL-C, tHcy and BP were higher in overweight or obese patients with PKU. By contrast, HDL-C was lower in these patients (Fig. 4). Levels of hsCRP were elevated in 4 out of the 55 analysed patients (7.27 %), all of them with overweight/obesity. No statistically significant differences between the underweight and the normal nutritional status groups were found, except from WC, which was lower in the group of patients with underweight and below 18 years of age (*p* = 0.007). With regard to 6R-BH_4_ treatment, statistically significant differences in HDL-C levels were observed between the 6R-BH_4_-treated and the non-treated group (mean: 60.65 ± 14.04 vs. 50.28 ± 11.61 mg/dL, *p* = 0.0367).

## Discussion

Reduction of animal origin lipids and cholesterol intake, which is one of the main characteristics of the PKU diet, could be considered to be the definition of non-atherogenic diet [12]. To support this hypothesis, previously published studies have suggested a similar or even improved lipid profile in PKU children compared to healthy controls [25]. Only few studies have explored lipid profile or other coronary heart disease-related parameters in PKU patients. Taking into account that PKU patients require a lifelong treatment, we consider that special attention and further investigations are required.

Our findings did not show significant differences in TGC levels between the PKU and MHPA groups of patients. Levels of total cholesterol (TC) in PKU children with good metabolic control have been assessed in few studies and their results have been inconclusive. Whereas in some of these studies have reported that PKU children on diet displayed lower TC levels compared with healthy controls [12, 26]; in one particular study there was no difference in TC levels between PKU and healthy children [13] and in another study TC levels were higher in patients with PKU [27]. In this present study, we showed that plasma TC levels were lower in the group of PKU patients than in the MHPA group. It should be pointed out that serum and tissue cholesterol concentrations depend on both a vegetarian diet, whose main sources of lipid are olive oil and cream cheese, and on cholesterogenesis [26, 28]. Furthermore, there are several regulatory mechanisms which contribute to cholesterol homeostasis. Although in this study we have only analyzed the blood lipid profile of patients with PKU, it is worth mentioning that an in vivo study, in which experimental hyperphenylalaninemia was induced in an animal model, demonstrated that there was an inhibition of two of the main regulatory enzymes of brain and liver cholesterogenesis: 3-hydroxy-3-methylglutaryl-CoA reductase and mevalonate-5-pyrophosphate decarboxylase [29]; and therefore a reduced cholesterol synthesis in the brain may indicate an association between impaired myelination and mental retardation in patients with PKU.

In contrast with other studies, which did not observe differences in plasma HDL levels in PKU children compared to controls [30–32], we found that HDL-C and ApoA levels were statistically significantly lower in PKU patients. However, LDL-C and ApoB levels were statistically significantly lower in the group of PKU patients, which is an indicator of low atherogenic risk. In addition, LDL-C/ApoB ratio was higher in PKU patients who had good dietary compliance. In this regard, it is worth mentioning that Campos et al.’s study [33] suggested that a high LDL-C/ApoB ratio usually correlates with the presence of larger and less atherogenic LDL particles, which are less susceptible to oxidative damage than small LDL particles. No clinical data of ischemic disease or other cardiovascular findings were observed in our patients, although it should be pointed out that only nine of them were between 40 and 50 years of age.

It has been previously reported that high serum Cu and low serum Zn concentrations were significantly associated with an increased mortality rate from all cardiovascular diseases and from coronary heart disease in particular [34]. In our study, we observed that only three patients had high Cu levels and 28 (19.9 %) of our patients had low serum Zn levels. Furthermore, a study in a rat animal model showed that low Cu levels were associated with the presence of larger LDLs and high serum CT, since this metal modulates the enzymatic activity of 3-hydroxy-3-methylglutaryl coenzyme A reductase, which is involved in cholesterol biosynthesis control [35].

Consistent with a previously published study [6], our data confirmed decreased plasma tHcy levels in PKU patients who have a good dietary compliance; and this was observed with methionine levels in the normal range for all the ages, including adult patients. In contrast, Schulpis et al.’ study observed that PKU patients on strict diet had moderate hyperhomocysteinaemia, which was probably due to the fact that these patients displayed low levels of vitamin B_12_ and folate, both essentials in Hcy metabolism [36].

Since it has been suggested that CRP levels could be considered to be an early endothelial dysfunction biomarker [37], we measured hsCRP, an inflammatory biomarker. We found elevated levels of hsCRP in 4 PKU adult patients with overweight/obesity.

Based on the fact that high blood pressure in adolescence or young adulthood is strongly related to later risk of stroke or coronary heart disease, independently of blood pressure in mid-life [30], in our present study, all patients displayed a statistically significant quadratic correlation between median Phe and systolic blood pressure and a statistically significant linear correlation between median Phe and diastolic blood pressure. It should be pointed out that only few studies have shown low diastolic blood pressure in patients with PKU on dietary treatment [14, 30]. This may be due to the fact that the evolution period was short and that studies on blood pressure in adults PKU are very scarce.

As far as overweight and obesity is concerned, there are several reports which suggested a clear tendency towards overweight and obesity in patients with PKU [32, 38–40], according to their BMI values. In this regard, we observed in our cohort that BMI Z-score and WC were higher in the group of PKU patients compared to the MHPA group. In fact, the AC, which is a strong indicator of caloric and protein sources because it reflects fat content and muscle, was elevated in 16.31 % of our PKU patients. Although a control group was not included in our study, when our results were compared to the nutritional status in the Spanish general population [41], we observed that the BMI Z-score is higher in our patients. Indeed, 37 % of our patients above 18 years of age had an elevated BMI Z-score, whereas in Spain it is a 26.7 % of the general population. In this line of research, Mazola et al. [42] showed similar BMI in patients with PKU compared with controls, but only 48 % of the patients in that study had good metabolic control. In our study, although a control group without PKU or MHPA was not included, we found that the BMI Z-score was statistically significantly higher in patients with good metabolic control compared to patients with poor metabolic control. This fact may be associated with protein substitutes intake and commercial low-Phe products, which most of them have a high caloric content.

Moreover, BMI Z-score significantly correlated with several cardiovascular risk factors such as elevated levels of TGC, tHcy and high BP. On the other hand, it was observed that patients under 6R-BH_4_ treatment, and therefore on a less restricted diet, had a clear tendency towards normal values of these parameters.

## Conclusions

Our findings demonstrated that PKU patients, who had good adherence to their diet, are not at risk of developing atherosclerosis because the levels of their lipids, lipoproteins and apoliproteins indicated a less atherogenic profile. Nevertheless, a high percentage of patients with low Phe tolerance and good dietary compliance were overweight or obese and showed elevated levels of atherogenic biochemical markers in addition to high tHcy levels and BP. These findings highlight the importance that in PKU patients it is safe to avoid overweight and obesity and advisable to monitor blood pressure, homocysteine and inflammatory biomarkers levels as well as others potential cardiovascular risk factors.

## Additional file

Additional file 1: Characteristics of Hyperphenylalaninaemia patients with altered values of the biochemical parameters studied. (DOCX 85 kb)

## Abbreviations

6R-BH_4_: (6R)-L-erythro-5,6,7,8-tetrahydrobiopterin
AC: Mid-upper arm circumference
ApoA: Apolipoprotein A
ApoB: Apolipoprotein B
B_12_: Vitamin B_12_
BMI: Body mass index
BP: Blood pressure
CPKU: Classic PKU
Cu: Cupper
DBP: Diastolic BP
tHcy: Total homocysteine
HDL-C: High-density lipoprotein cholesterol
HPA: Hyperphenylalaninaemia
hsCRP: High sensitivity C-Reactive Protein
LDL-C: Low density lipoprotein-cholesterol
Met: Methionine
MHPA: Mild-hyperphenylalaninaemia
MPKU: Mild-moderate PKU
Phe: Phenylalanine
PKU: Phenylketonuria
SBP: Systolic BP
TC: Total cholesterol
TGC: Triglycerides
WC: Waist circumference
Zn: Zinc
