# Supplementary material for: Lipid profile status and other related factors in patients with Hyperphenylalaninaemia
Source: Orphanet J Rare Dis. 2016 Sep 9;11(1):123. doi: 10.1186/s13023-016-0508-x (PMC5016957; doi:10.1186/s13023-016-0508-x)
Supplement: Additional file 1: — Characteristics of Hyperphenylalaninaemia patients with altered values of the biochemical parameters studied. (DOCX 85 kb) [file 13023_2016_508_MOESM1_ESM.docx]

| P | Sex | Age | BMI | WC | AC | Diag | E/L Diag | BH4 Treat | Phe Tol.  mg/day | Phe med.  µmol/L | hsCRP  mg/dL | Total Chol.  mg/dL | HDL  mg/dL | LDL  mg/dL | ApoA  mg/dL | ApoB  mg/dL | LDL/ ApoB | Trig.  mg/dL | tHcy.  µmol/L | Met  µmol/L | Syst BP  mmHg | Diast BP  mmHg | B12  pg/mL | Zn  µg/dL | Cu  µg/dL | Zn/ Cu |
| --- | --- | --- | --- | --- | --- | --- | --- | --- | --- | --- | --- | --- | --- | --- | --- | --- | --- | --- | --- | --- | --- | --- | --- | --- | --- | --- |
| 1 | M | 10y | N | ↑↑ | ↑ | CPKU | E | NO | 352↓ | 171 | 0.04 | 106↓ | 53 | 42 | 141 | 36 | 1.17 | 58 | 5 | 24 | 100 | 66 | 726 | 55↓ | 78 | 0.71 |
| 2 | M | 12y 7m | N | N | ↑ | CPKU | E | NO | 148↓ | 476 | - | 100↓ | 48 | 44 | 143 | 40 | 1.1 | 41 | 5 | 23 | 107 | 66 | 471 | 69 | 66 | 1.05 |
| 3 | F | 16y 6m | N | N | N | CPKU | E | NO | 314↓ | 388 | - | 136 | 50 | 75 | 150 | 57 | 1.32 | 56 | 4↓ | 24 | 108 | 62 | 749 | 60↓ | 62↓ | 0.97 |
| 4 | M | 8y 10m | N | N | N | CPKU | E | NO | 397↓ | 160 | - | 149 | 50 | 81 | 158 | 66 | 1.23 | 92 | 5 | 21 | 92 | 59 | 1136 | 55↓ | 74 | 0.74 |
| 5 | M | 2y 6m | N | ↑ | ↑↑ | CPKU | E | NO | 259↓ | 222 | 0.02 | 175 | 37↓ | 116 | 180 | 97 | 1.2 | 55 | 5 | 30 | 117 | 66 | 464 | 99 | 82 | 1.21 |
| 6 | F | 44y 4m | ↑↑ | ↑↑ | ↑ | CPKU | L | NO | 400↓ | 900↑ | - | 197 | 43 | 136↑ | 163 | 108 | 1.26 | 214↑ | 6 | 28 | 138↑ | 100↑ | 282 | 91 | 67 | 1.36 |
| 7 | F | 23y 2m | ↑↑ | ↑ | ↑ | CPKU | E | NO | 237↓ | 370 | - | 145 | 47 | 88 | 143 | 64 | 1.38 | 51 | 5 | 27 | 123 | 73 | 673 | 89 | 139 | 0.64 |
| 8 | F | 14y 1m | N | ↓ | N | CPKU | E | NO | 225↓ | 363 | - | 118↓ | 40 | 63 | 140 | 54 | 1.17 | 75 | 3↓ | 26 | 109 | 63 | 774 | 87 | 67 | 1.3 |
| 9 | F | 50y 8m | ↑↑ | ↑↑ | ↑ | CPKU | L | NO | 360↓ | 900↑ | - | 193 | 46 | 102 | - | - | - | 227↑ | 6 | 24 | 124 | 86 | 558 | 97 | 93 | 1.04 |
| 10 | M | 15y 7m | N | N | N | CPKU | E | NO | 373↓ | 304 | 0.13 | 144 | 53 | 79 | - | - | - | 62 | 7 | 32 | 119 | 68 | 615 | 72 | 70 | 1.03 |
| 11 | F | 15y 4m | N | N | N | CPKU | E | NO | 383↓ | 227 | - | 108↓ | 41 | 49 | 122 | 40 | 1.23 | 42 | 3↓ | 27 | 99 | 74 | 1038 | 101 | 73 | 1.38 |
| 12 | F | 16y 4m | N | ↑↑ | N | CPKU | E | NO | 295↓ | 237 | - | 123 | 43 | 63 | - | 57 | 1.11 | 87 | 8 | 35 | 122 | 86 | 512 | 91 | 72 | 1.26 |
| 13 | F | 40y | ↑↑ | ↑↑ | N | CPKU | L | NO | 280↓ | 600 | - | 140 | 36↓ | 80 | 138 | 76 | 1.05 | 121 | 7 | 31 | 104 | 68 | 473 | 74 | 80 | 0.92 |
| 14 | M | 34y 8m | ↑↑ | ↑↑ | ↑↑ | CPKU | L | NO | 300↓ | 287 | - | 161 | 48 | 90 | 145 | 84 | 1.07 | 115 | 2↓ | 24 | 120 | 69 | 966 | 97 | 67 | 1.45 |
| 15 | M | 32y 7m | ↑ | N | N | CPKU | E | NO | 330↓ | 840↑ | - | 141 | 47 | 85 | - | - | - | 70 | 7 | 31 | 115 | 62 | 516 | 87 | 83 | 1.05 |
| 16 | F | 28y 4m | ↑ | ↑ | ↑ | CPKU | E | NO | 270↓ | 570 | - | 151 | 57 | 76 | - | - | - | 92 | 6 | 30 | 130 | 78 | 559 | 67 | 68 | 0.99 |
| 17 | F | 37y 7m | N | N | N | CPKU | E | NO | 378↓ | 490 | - | 162 | 49 | 99 | - | - | - | 75 | 7 | 29 | 127 | 80 | 182 | 71 | 76 | 0.93 |
| 18 | M | 21y 8m | ↑ | ↑ | N | CPKU | E | NO | 411↓ | 613↑ | - | 95↓ | 38↓ | 51 | - | - | - | 96 | 7 | 28 | 125 | 70 | 650 | - | 85 | - |
| 19 | F | 28y 6m | ↑ | ↑ | ↑ | CPKU | E | NO | 229↓ | 388 | - | 177 | 50 | 70 | - | - | - | 72 | 5.5 | 32 | 129 | 64 | 496 | 52↓ | 52↓ | 1 |
| 20 | M | 41y 10m | ↑ | ↑ | ↑ | CPKU | L | NO | 555 | 816↑ | 1.09 | 127 | 46 | 64 | - | - | - | 122 | 9 | - | 143↑ | 95↑ | 743 | 83 | 70 | 1.19 |
| 21 | F | 6y 3m | N | ↑↑ | ↑↑ | CPKU | E | NO | 264↓ | 169 | 0.03 | 135 | 47 | 75 | 131 | 54 | 1.39 | 45 | 2↓ | 22 | 110 | 58 | 1034 | 68 | 91 | 0.75 |
| 22 | F | 22y 10m | ↑↑ | N | ↑ | CPKU | E | YES | 237↓ | 370 | 1.15↑ | 145 | 47 | 88 | 143 | 64 | 1.38 | 51 | 5 | 28 | 104 | 74 | 673 | 89 | 139 | 0.64 |
| 23 | M | 4y | N | ↑↑ | ↑↑ | CPKU | E | YES | 327↓ | 430↑ | 0.03 | 130 | 70 | 61 | 144 | 49 | 1.24 | 45 | 7 | 27 | 112 | 77 | 824 | 69 | 62↓ | 1.11 |
| 24 | F | 3y 1m | N | N | N | CPKU | E | YES | 319↓ | 98 | 0.02 | 111↓ | 79 | 62 | 96↓ | 63 | 0.98 | 86 | 4↓ | 29 | - | - | 1349 | 97 | 110 | 0.88 |
| 25 | F | 50y 7m | ↑↑ | ↑↑ | N | CPKU | E | NO | 340↓ | 287 | - | 193 | 46 | 102 | - | - | - | 187↑ | 6 | 25 | 124 | 86 | 558 | 97 | 93 | 1.04 |
| 26 | F | 42y 1m | ↑ | ↑ | - | CPKU | E | NO | 121↓ | 279 | 1.22↑ | 189 | 42 | 116 | - | - | - | 154↑ | 5.5 | 27 | - | - | 660 | 69.2 | 62↓ | 1.12 |
| 27 | M | 17y | N | ↑ | - | CPKU | E | NO | 210↓ | 195 | - | 127 | 62 | 53 | - | - | - | 61 | 7.91 | 33 | 120 | 60 | 361 | 113 | 69 | 1.64 |
| 28 | F | 18y 2m | ↓ | ↑ | - | CPKU | E | NO | 256↓ | 756↑ | 0.02 | 145 | 74 | 63 | - | - | - | 42 | 4.86↓ | 31 | - | - | 858 | 76 | 75 | 1.01 |
| 29 | F | 35y 10m | ↑ | N | - | CPKU | E | NO | 435↓ | 294 | 0.11 | 195 | 53 | 125 | - | - | - | 85 | 5.38 | 30 | - | - | 2000↑ | 71 | 64↓ | 1.11 |
| 30 | F | 4y 4m | N | N | - | CPKU | L | NO | 401↓ | 735↑ | 0.02 | 149 | 50 | 93 | - | - | - | 30 | 3.75↓ | 25 | - | - | 737 | 73 | 61↓ | 1.2 |
| 31 | F | 23y 2m | ↑ | ↑ | - | CPKU | L | NO | 360↓ | 516 | 1.24↑ | 160 | 47 | 89 | - | - | - | 118 | 9.01 | 29 | - | - | 209 | 81 | 74 | 1.09 |
| 32 | M | 43y | ↑ | ↑ | - | CPKU | E | NO | 626 | 132 | 0.02 | 194 | 59 | 112 | - | - | - | 116 | 6.09 | 31 | - | - | 368 | 75 | 61↓ | 1.23 |
| 33 | F | 9y 2m | N | N | - | CPKU | L | NO | 245↓ | 1272↑ | 0.02 | 135 | 49 | 78 | 137 | 70 | 1.11 | 40 | 4.19↓ | 22 | - | - | 616 | 85 | 88 | 0.97 |
| 34 | M | 23y 2m | N | N | - | CPKU | E | NO | 452↓ | 310 | 0.02 | 141 | 51 | 76 | 132 | 66 | 1.15 | 72 | 25.66↑ | 20 | - | - | 362 | 81.2 | 62↓ | 1.31 |
| 35 | M | 26y 7m | ↓ | N | - | CPKU | E | NO | 303↓ | 320 | 0.04 | 101↓ | 47 | 47 | - | - | - | 33 | 4.03↓ | 27 | - | - | 632 | 96 | 64↓ | 1.5 |
| 36 | M | 9m | N | N | - | CPKU | E | NO | 303↓ | 92 | - | 155 | 51 | 79 | 138 | 71 | 1.11 | 123↑ | 2.5↓ | 23 | 85 | 37 | 635 | 175↑ | 63↓ | 2.78 |
| 37 | M | 1y 1m | N | N | - | CPKU | E | NO | 325↓ | 384↑ | - | 93↓ | 29↓ | 48 | 92↓ | 48 | 1 | 80 | 2.07↓ | 24 | 98 | 65 | 1548 | 81 | 109 | 0.74 |
| 38 | M | 1y 9m | N | N | - | CPKU | E | NO | 250↓ | 90 | - | 111↓ | 37↓ | 57 | 110↓ | 67 | 0.85 | 81 | 5.45 | 28 | 87 | 40 | 869 | 68 | 60↓ | 1.13 |
| 39 | F | 3y | N | ↓ | ↓ | CPKU | E | NO | 252↓ | 126 | - | 99↓ | 43 | 46 | 122 | 40 | 1.15 | 48 | 5.06 | 21 | 117 | 78 | 826 | 73 | 89 | 0.82 |
| 40 | M | 3y 6m | ↓ | ↓ | ↓ | CPKU | E | NO | 473↓ | 348 | - | 129 | 54 | 66 | 133 | 47 | 1.4 | 45 | 3.77↓ | 24 | 89 | 65 | 375 | 63↓ | 90 | 0.7 |
| 41 | M | 3y 8m | N | N | ↓ | CPKU | E | NO | 300↓ | 102 | 0.02 | 159 | 50 | 95 | - | - | - | 63 | 5.55 | 27 | 112 | 65 | 731 | 60↓ | 115 | 0.52 |
| 42 | F | 3y 8m | N | ↑↑ | ↑↑ | CPKU | E | NO | 339↓ | 156 | 0.02 | 175 | 65 | 89 | 179 | 79 | 1.13 | 108↑ | 4.79↓ | 26 | 114 | 59 | 577 | 60↓ | 108 | 0.56 |
| 43 | F | 4y 3m | ↓ | N | ↓ | CPKU | E | NO | 289↓ | 225 | 0.03 | 175 | 55 | 105 | 168 | 71 | 1.48 | 77 | 4.34↓ | 22 | 103 | 59 | 830 | 60↓ | 60↓ | 1 |
| 44 | M | 3y 10m | N | ↑↑ | N | CPKU | E | NO | 236↓ | 288 | 0.02 | 162 | 42 | 101 | 123 | 76 | 1.33 | 102↑ | 4.49↓ | 28 | 107 | 69 | 503 | 80 | 93 | 0.86 |
| 45 | F | 4y 6m | N | N | ↓ | CPKU | E | NO | 345↓ | 150 | 0.03 | 127 | 42 | 52 | 134 | 51 | 1.02 | 173↑ | 3.4↓ | 25 | 90 | 52 | 921 | 57↓ | 104 | 0.55 |
| 46 | M | 4y 3m | ↓ | ↑ | ↑↑ | CPKU | E | NO | 464↓ | 348 | 0.02 | 126 | 51 | 62 | 139 | 48 | 1.29 | 66 | 3.63↓ | 29 | 102 | 72 | 777 | 53↓ | 89 | 0.6 |
| 47 | M | 4y 4m | N | N | N | CPKU | E | NO | 315↓ | 228 | 0.02 | 115↓ | 19↓ | 70 | 84↓ | 78 | 0.9 | 135↑ | 3.18↓ | 27 | 98 | 65 | 1481 | 111 | 109 | 1.02 |
| 48 | M | 5y 9m | N | N | N | CPKU | E | NO | 379↓ | 207 | 0.04 | 159 | 61 | 90 | 160 | 66 | 1.36 | 40 | 3.17↓ | 26 | 110 | 70 | 483 | 99 | 80 | 1.24 |
| 49 | F | 7y 4m | N | N | ↓ | CPKU | E | NO | 422↓ | 276 | 0.03 | 175 | 39↓ | 118 | 112↓ | 87 | 1.36 | 89 | 4.01↓ | 25 | 111 | 72 | 1010 | 75 | 119 | 0.63 |
| 50 | M | 7y | N | N | N | CPKU | E | NO | 403↓ | 282 | 0.02 | 168 | 48 | 105 | 140 | 70 | 1.5 | 128↑ | 4.99↓ | 28 | 98 | 57 | 568 | 78 | 116 | 0.67 |
| 51 | F | 10y 6m | N | N | ↓ | CPKU | E | NO | 418↓ | 300 | - | 165 | 46 | 101 | 143 | 66 | 1.53 | 94 | 5.83 | 29 | 108 | 62 | 594 | 43↓ | 75 | 0.57 |
| 52 | M | 11y 2m | N | ↓ | ↓ | CPKU | E | NO | 55↓ | 162 | - | 110↓ | 49 | 53 | 133 | 44 | 1.2 | 42 | 4↓ | 23 | 105 | 74 | 760 | 84 | 73 | 1.15 |
| 53 | F | 18y 1m | ↑↑ | ↑↑ | ↑↑ | CPKU | L | NO | 168↓ | 228 | - | 171 | 38↓ | 88 | 134 | 92 | 0.96 | 224↑ | 4.25↓ | 25 | 128 | 82 | 525 | 107 | 100 | 1.07 |
| 54 | M | 19y 3m | N | N | ↓ | CPKU | E | NO | 469↓ | 378 | - | 113↓ | 40 | 53 | 129 | 47 | 1.13 | 102 | 7.45 | - | 110 | 58 | 756 | 150↑ | 74 | 2.03 |
| 55 | M | 19y 10m | ↑ | N | ↓ | CPKU | L | NO | 503 | 252 | - | 144 | 39↓ | 83 | 137 | 70 | 1.19 | 112 | 5.01 | - | 115 | 60 | 840 | 97 | 36↓ | 2.69 |
| 56 | M | 20y 8m | ↑ | ↑ | - | CPKU | L | NO | 217↓ | 378 | - | 119↓ | 36↓ | 64 | 128 | 48 | 1.33 | 98 | 6.84 | - | 125 | 76 | 936 | 95 | 64↓ | 1.48 |
| 57 | M | 22y 7m | ↑ | ↑ | - | CPKU | E | NO | 429↓ | 516 | - | 181 | 50 | 112 | 141 | 66 | 1.7 | 98 | 5.44 | 27 | 105 | 79 | 997 | 71 | 70 | 1.01 |
| 58 | F | 23y 3m | N | N | - | CPKU | E | NO | 440↓ | 522 | - | 273↑ | 93 | 157↑ | 263 | 122↑ | 1.29 | 116 | 4.51↓ | 26 | 134 | 78 | 315 | 63↓ | 128 | 0.49 |
| 59 | M | 24y 1m | ↑ | ↑ | - | CPKU | E | NO | 305↓ | 840↑ | - | 127 | 49 | 65 | 168 | 58 | 1.12 | 64 | 9.63 | - | 138↑ | 90↑ | 946 | 74 | 77 | 0.96 |
| 60 | F | 24y 9m | ↑ | ↑ | - | CPKU | E | NO | 490↓ | 1500↑ | - | 124 | 50 | 69 | 132 | 45 | 1.53 | 27↓ | 5.66 | 31 | 119 | 82 | 388 | 100 | 38↓ | 2.63 |
| 61 | F | 25y 1m | ↑ | ↑↑ | - | CPKU | E | NO | 221↓ | 600 | - | 199 | 42 | 127 | 138 | 110↑ | 1.15 | 157↑ | 6.71 | 30 | 127 | 76 | 776 | 111 | 73 | 1.52 |
| 62 | F | 28y 9m | N | N | - | CPKU | E | NO | 432↓ | 162 | - | 119↓ | 41 | 58 | 137 | 51 | 1.14 | 103 | 4.39↓ | 27 | 135 | 75 | 2000↑ | 121 | 65 | 1.86 |
| 63 | M | 29y 10m | ↑ | ↑ | - | CPKU | E | NO | 490↓ | 1140↑ | - | 134 | 43 | 66 | 147 | 69 | 0.96 | 130 | 17.99↑ | 29 | 125 | 81 | 237 | 112 | 43↓ | 2.6 |
| 64 | F | 31y 1m | N | ↑ | - | CPKU | E | NO | 471↓ | 840↑ | - | 121 | 51 | 56 | 134 | 46 | 1.22 | 124 | 14.4 | 26 | 117 | 58 | 384 | 45↓ | 57↓ | 0.79 |
| 65 | F | 35y 9m | ↑↑ | ↑↑ | - | CPKU | L | NO | 323↓ | 1200↑ | - | 165 | 64 | 93 | 190 | 55 | 1.69 | 47 | 10.94 | - | 141↑ | 90↑ | 398 | 128 | 106 | 1.21 |
| 66 | M | 40y | ↑ | ↑ | - | CPKU | L | NO | 360↓ | 600 | - | 158 | 44 | 96 | 139 | 70 | 1.37 | 92 | 11.43 | 31 | 133 | 83 | 332 | 102 | 57↓ | 1.79 |
| 67 | F | 17y 10m | N | N | ↓ | MPKU | E | YES | 2585 | 277 | 0.01 | 185 | 92 | 81 | 206 | 80 | 1.01 | 58 | 4↓ | 26 | 118 | 70 | 696 | 65 | 65 | 1 |
| 68 | M | 5y | N | N | N | MPKU | E | NO | 405↓ | 76 | - | 125 | 51 | 47 | 154 | 54 | 0.87 | 109↑ | 3↓ | 22 | 104 | 58 | 1187 | 64↓ | 71 | 0.9 |
| 69 | F | 9y 8m | N | N | N | MPKU | E | YES | 872 | 161 | 0.02 | 193 | 52 | 127 | 142 | 99 | 1.28 | 70 | 4↓ | 25 | 110 | 63 | 950 | 60↓ | 86 | 0.7 |
| 70 | F | 9y 8m | N | N | N | MPKU | E | YES | 900 | 155 | - | 184 | 56 | 120 | 149 | 91 | 1.32 | 42 | 4↓ | 29 | 108 | 43 | 878 | 65 | 79 | 0.82 |
| 71 | F | 7y 4m | N | ↑ | N | MPKU | E | YES | 815 | 266 | - | 123 | 63 | 51 | 157 | 47 | 1.09 | 43 | 4↓ | 30 | 99 | 58 | 415 | 68 | 76 | 0.89 |
| 72 | F | 21y 7m | N | N | N | MPKU | E | YES | 700 | 262 | - | 183 | 77 | 92 | - | - | - | 88 | 5 | 25 | 133 | 81 | 596 | 76 | 86 | 0.88 |
| 73 | F | 12y 1m | N | N | N | MPKU | E | NO | 550 | 196 | 0.02 | 147 | 61 | 74 | - | - | - | 133↑ | 3↓ | 22 | 117 | 61 | 463 | 81 | 50↓ | 1.62 |
| 74 | F | 34y 6m | ↑↑ | ↑↑ | ↑ | MPKU | L | NO | 530 | 310 | - | 237↑ | 55 | 147↑ | - | - | - | 207↑ | 10 | - | 163↑ | 111↑ | 256 | 72 | 157↑ | 0.46 |
| 75 | F | 40y 10m | ↑↑ | ↑↑ | ↑ | MPKU | L | NO | 612 | 766↑ | - | 159 | 43 | 60 | - | - | - | 129 | 8 | 26 | 125 | 67 | 333 | 48↓ | 68 | 0.71 |
| 76 | M | 28y 6m | ↑ | ↑↑ | ↑ | MPKU | E | YES | 800 | 452 | - | 182 | 51 | 88 | - | - | - | 112 | 5 | 24 | 128 | 68 | 571 | 84 | 72 | 1.17 |
| 77 | F | 6y 6m | N | ↓ | N | MPKU | E | YES | 1050 | 195 | - | 142 | 50 | 85 | 153 | 73 | 1.16 | 37 | 5 | - | 121↑ | 89↑ | 868 | 63↓ | 92 | 0.68 |
| 78 | F | 12y 9m | N | N | N | MPKU | E | YES | 1600 | 245 | - | 146 | 73 | 67 | 184 | 53 | 1.26 | 28↓ | 6 | 31 | 109 | 47 | 698 | 59↓ | 63↓ | 0.94 |
| 79 | F | 28y 4m | ↑ | ↑↑ | N | MPKU | E | NO | 600 | 290 | - | 188 | 45 | 113 | 151 | 104 | 1.09 | 150↑ | 9 | - | 139↑ | 98↑ | 314 | 66 | 76 | 0.87 |
| 80 | M | 12y 2m | N | N | - | MPKU | E | YES | 823.8 | 222 | 0.08 | 125 | 41 | 63 | - | - | - | 106 | 5.1 | 32 | - | - | 389 | 74.3 | 70 | 1.06 |
| 81 | F | 10y 2m | N | N | - | MPKU | E | YES | 922 | 198 | 0.1 | 156 | 43 | 89 | - | - | - | 120 | 2.33↓ | 21 | - | - | 437 | 70 | 60↓ | 1.17 |
| 82 | M | 5y 1m | N | N | - | MPKU | E | YES | 1069 | 117 | - | 151 | 68 | 71 | - | - | - | 62 | 3.52↓ | 23 | - | - | 501 | 87.3 | 68 | 1.28 |
| 83 | M | 3y 9m | N | N | - | MPKU | E | NO | 503 | 300 | 0.02 | 165 | 53 | 95 | - | - | - | 84 | 4.1↓ | 24 | - | - | 766 | 67.2 | 62↓ | 1.08 |
| 84 | F | 26y 2m | ↑ | ↑↑ | - | MPKU | E | YES | 810 | 222 | 3.98↑ | 181 | 53 | 84 | - | - | - | 104 | 6.13 | 31 | - | - | 581 | 78 | 65 | 1.2 |
| 85 | F | 10y 2m | N | N | - | MPKU | E | NO | 622 | 990↑ | 0.1 | 156 | 43 | 89 | - | - | - | 120 | 2.33↓ | 21 | 100 | 57 | 437 | 70 | 73 | 0.96 |
| 86 | M | 42y | ↑ | ↑ | - | MPKU | E | NO | 555 | 180 | 0.09 | 160 | 37↓ | 106 | - | - | - | 87 | 9.09 | 25 | - | - | 366 | 116 | 64↓ | 1.81 |
| 87 | M | 11y 2m | N | ↑ | - | MPKU | E | YES | 2052 | 150 | 0.32 | 174 | 56 | 104 | - | - | - | 71 | 4.76↓ | 23 | 115 | 57 | 420 | 98.2 | 63↓ | 1.56 |
| 88 | F | 2y 10m | N | N | - | MPKU | E | NO | 386↓ | 882↑ | 0.02 | 170 | 30↓ | 116 | - | - | - | 122↑ | 3.49↓ | 32 | - | - | 669 | 108 | 95 | 1.14 |
| 89 | M | 19y 7m | ↑ | ↑↑ | - | MPKU | E | NO | 412↓ | 174 | 0.02 | 145 | 38↓ | 95 | - | - | - | 60 | 8.54 | 31 | - | - | 387 | 95 | 65 | 1.46 |
| 90 | M | 12y | N | N | - | MPKU | E | NO | 438↓ | 192 | 0.02 | 105↓ | 57 | 39 | - | - | - | 45 | 9.17 | 33 | - | - | 290 | 68 | 59↓ | 1.15 |
| 91 | M | 8y 3m | N | N | - | MPKU | E | NO | 587 | 264 | 0.02 | 165 | 70 | 76 | 156 | 72 | 1.06 | 96 | 5.4 | 19 | - | - | 582 | 65 | 60↓ | 1.08 |
| 92 | M | 4y 9m | N | N | ↑ | MPKU | E | NO | 255↓ | 408↑ | - | 116↓ | 43 | 59 | 125 | 56 | 1.05 | 72 | 5.84 | 21 | 86 | 41 | 617 | 83 | 115 | 0.72 |
| 93 | M | 6y 8m | N | ↑↑ | ↑↑ | MPKU | E | NO | 272↓ | 330 | - | 181 | 47 | 103 | 160 | 89 | 1.16 | 156↑ | 10.61 | 30 | 117 | 70 | 442 | 92 | 106 | 0.87 |
| 94 | M | 7y 1m | N | N | ↓ | MPKU | E | NO | 258↓ | 336 | - | 162 | 53 | 95 | 148 | 60 | 1.58 | 126↑ | 3.74↓ | 24 | 108 | 78 | 487 | 84 | 83 | 1.01 |
| 95 | M | 8y 3m | N | N | N | MPKU | E | YES | 1829 | 425 | - | 142 | 60 | 69 | 168 | 45 | 1.53 | 32 | 4.48↓ | 26 | 112 | 58 | 788 | 91 | 83 | 1.1 |
| 96 | F | 13y 9m | N | ↑↑ | ↓ | MPKU | E | NO | 266↓ | 450 | - | 146 | 34↓ | 82 | 119 | 71 | 1.15 | 152↑ | 4.26↓ | 25 | 108 | 57 | 347 | 78 | 78 | 1 |
| 97 | M | 14y 4m | N | N | ↓ | MPKU | E | NO | 425↓ | 246 | - | 159 | 49 | 101 | 134 | 63 | 1.6 | 46 | 7.22 | 28 | 116 | 57 | 464 | 102 | 71 | 1.44 |
| 98 | F | 15y 9m | N | N | N | MPKU | E | NO | 412↓ | 516 | - | 114↓ | 46 | 55 | 132 | 46 | 1.2 | 67 | 5.55 | 20 | 105 | 59 | 505 | 46↓ | 70 | 0.66 |
| 99 | F | 16y 7m | N | N | ↓ | MPKU | E | NO | 447↓ | 450 | - | 108↓ | 42 | 53 | 148 | 52 | 1.02 | 61 | 9.75 | 23 | 121 | 75 | 331 | 85 | 72 | 1.18 |
| 100 | F | 28y 2m | ↑↑ | ↑↑ | - | MPKU | E | NO | 467↓ | 620↑ | - | 175 | 40 | 94 | 148 | 91 | 1.03 | 198↑ | 4.6↓ | 26 | 120 | 80 | 1280 | 76 | 75 | 1.01 |
| 101 | F | 10y 1m | N | N | ↑ | MHPA | E | NO | - | 115 | 0.02 | 175 | 57 | 109 | 160 | 90 | 1.21 | 43 | 9 | 29 | 106 | 65 | 308 | 59↓ | 84 | 0.7 |
| 102 | F | 20y 1m | ↑ | N | N | MHPA | E | NO | - | 165 | - | 174 | 64 | 87 | 207 | 81 | 1.07 | 115 | 7 | 25 | 115 | 63 | 256 | 61↓ | 145↑ | 0.42 |
| 103 | F | 4y 9m | N | N | N | MHPA | E | NO | 900 | 111 | 0.02 | 174 | 66 | 102 | 163 | 77 | 1.32 | 30 | 4↓ | 29 | 100 | 64 | 490 | 73 | 76 | 0.96 |
| 104 | F | 5y 3m | N | N | N | MHPA | E | NO | 1100 | 198 | - | 175 | 52 | 109 | 152 | 88 | 1.24 | 68 | 4↓ | 28 | 92 | 56 | 416 | 87 | 78 | 1.12 |
| 105 | F | 17y 9m | N | N | N | MHPA | E | NO | - | 130 | - | 163 | 38↓ | 86 | - | - | - | 195↑ | 11 | 21 | 105 | 63 | 684 | 72 | 73 | 0.99 |
| 106 | M | 8y 4m | N | ↑↑ | ↑↑ | MHPA | E | NO | - | 180 | - | 124 | 53 | 56 | - | - | - | 77 | 4↓ | 21 | ↑130 | ↑80 | 423 | 75 | 86 | 0.87 |
| 107 | M | 5y 10m | N | ↓ | N | MHPA | E | NO | 790 | 175 | - | 229↑ | 82 | 134↑ | 187 | 113↑ | 1.19 | 65 | 3↓ | 20 | 107 | 70 | 778 | 64↓ | 83 | 0.77 |
| 108 | F | 10y 8m | N | N | N | MHPA | E | NO | - | 155 | 0.01 | 170 | 42 | 106 | 115 | 86 | 1.23 | 111 | 6 | 27 | 93 | 68 | 382 | 64↓ | 50↓ | 1.28 |
| 109 | M | 9y 2m | N | N | N | MHPA | E | NO | - | 210 | - | 145 | 54 | 77 | 139 | 63 | 1.22 | 71 | 2↓ | 22 | 110 | 77 | 985 | 63↓ | 62↓ | 1.02 |
| 110 | F | 5y 2m | ↓ | ↓ | N | MHPA | E | NO | - | 260 | - | 169 | 50 | 109 | 134 | 85 | 1.28 | 49 | 3↓ | 24 | 104 | 74 | 911 | 70 | 98 | 0.71 |
| 111 | M | 11y 10m | N | ↑ | N | MHPA | E | NO | 1100 | 198 | - | 145 | 70 | 67 | - | - | - | 42 | 6 | 27 | 97 | 68 | - | 66 | 64↓ | 1.03 |
| 112 | M | 3y 1m | N | N | - | MHPA | E | NO | 880 | 320 | - | 130 | 38↓ | 78 | 113↓ | 70 | 1.11 | 70 | 4↓ | 24 | 95 | 65 | 607 | 78 | 93 | 0.84 |
| 113 | F | 7y 3m | ↓ | N | N | MHPA | E | NO | 2601 | 258 | 0.02 | 152 | 51 | 85 | 141 | 49 | 1.73 | 68 | 6 | 26 | - | - | 506 | 73 | 71 | 1.03 |
| 114 | M | 11y 9m | N | ↑↑ | ↑↑ | MHPA | E | NO | 2300 | 280 | - | 184 | 59 | 110 | 169 | 85 | 1.29 | 74 | 7 | 27 | 101 | 65 | 693 | 65 | 97 | 0.67 |
| 115 | F | 10y 2m | N | N | N | MHPA | E | NO | 1500 | 240 | - | 206↑ | 89 | 106 | 189 | 86 | 1.23 | 54 | 5 | 28 | 97 | 62 | 829 | 68 | 84 | 0.81 |
| 116 | F | 4y 2m | N | N | N | MHPA | E | NO | 1900 | 190 | - | 155 | 50 | 95 | 136 | 76 | 1.25 | 52 | 8 | 21 | 109 | 76 | 565 | 71 | 94 | 0.76 |
| 117 | F | 6y 8m | ↓ | ↓ | N | MHPA | E | NO | - | 242 | - | 159 | 84 | 67 | 200 | 57 | 1.18 | 41 | 6 | 29 | 109 | 77 | 696 | 65 | 74 | 0.88 |
| 118 | F | 46y 1m | N | N | - | MHPA | E | NO | 888 | 235 | - | 188 | 45 | 92 | 185 | 65 | 1.42 | 116 | 8 | 31 | 105 | 75 | 468 | 69 | 66 | 1.05 |
| 119 | F | 3y | N | N | - | MHPA | L | NO | 1521 | 363↑ | 0.03 | 145 | 45 | 87 | - | - | - | 64 | 4.56↓ | 30 | - | - | 967 | 98 | 79 | 1.24 |
| 120 | M | 6m | N | N | - | MHPA | E | NO | 820 | 210 | - | 206↑ | 60 | 128 | 177 | 96 | 1.33 | 92 | 8.03 | 37 | 83 | 42 | 361 | 95 | 74 | 1.28 |
| 121 | M | 1y | N | N | - | MHPA | E | NO | 1130 | 186 | - | 151 | 46 | 81 | 134 | 67 | 1.21 | 122↑ | 3.09↓ | 23 | 85 | 38 | 536 | 72 | 104 | 0.69 |
| 122 | M | 1y 1m | N | N | - | MHPA | E | NO | 2064 | 348 | - | 169 | 63 | 96 | - | - | - | 48 | 6 | 28 | 117 | 72 | - | 67 | 58↓ | 1.16 |
| 123 | M | 1y 10m | N | N | - | MHPA | E | NO | 1568 | 246 | 0.02 | 137 | 47 | 78 | 137 | 57 | 1.37 | 62 | 4.51↓ | 27 | 90 | 42 | 470 | 123 | 120 | 1.02 |
| 124 | F | 2y 2m | N | ↑↑ | ↑ | MHPA | E | NO | 2590 | 162 | 0.02 | 153 | 46 | 96 | 132 | 78 | 1.23 | 59 | 3.17↓ | 22 | 99 | 66 | 691 | 65 | 70 | 0.93 |
| 125 | M | 4y 4m | N | N | N | MHPA | E | NO | 1400 | 306 | 0.02 | 135 | 55 | 69 | 142 | 56 | 1.23 | 54 | 7.13 | 29 | 109 | 65 | 558 | 248↑ | 83 | 2.99 |
| 126 | M | 5y 1m | N | ↓ | N | MHPA | E | NO | 1952 | 198 | 0.01 | 176 | 68 | 98 | 169 | 73 | 1.34 | 48 | 4.97↓ | 25 | 108 | 67 | 700 | 62↓ | 95 | 0.65 |
| 127 | F | 5y 10m | N | N | N | MHPA | E | NO | 2033 | 234 | 0.06 | 147 | 60 | 77 | 156 | 61 | 1.26 | - | 5.58 | 24 | 108 | 68 | 749 | 64↓ | 79 | 0.81 |
| 128 | F | 6y | ↓ | N | ↓ | MHPA | E | NO | 1328 | 120 | - | 142 | 46 | 82 | 134 | 57 | 1.44 | 72 | 5.54 | 22.5 | 110 | 70 | 607 | 69 | 96 | 0.72 |
| 129 | F | 6y 3m | N | N | N | MHPA | E | NO | 2634 | 222 | - | 162 | 43 | 100 | 189 | 95 | 1.05 | - | 4.51↓ | 21.7 | 111 | 62 | 928 | 100 | 95 | 1.05 |
| 130 | M | 5y 10m | N | N | N | MHPA | E | NO | 1099 | 258 | 0.03 | 149 | 71 | 71 | 163 | 61 | 1.16 | 40 | 3.83↓ | 26 | 102 | 71 | 676 | 137 | 78 | 1.76 |
| 131 | M | 6y 1m | N | N | N | MHPA | E | NO | 3594 | 180 | - | 163 | 57 | 91 | 157 | 59 | 1.54 | 81 | 6 | 29 | 92 | 46 | 536 | 63↓ | 66 | 0.95 |
| 132 | M | 6y 8m | ↓ | N | ↓ | MHPA | E | NO | 3502 | 132 | - | 207↑ | 51 | 120 | 197 | 118↑ | 1.02 | - | 6.48 | 28 | 96 | 56 | 916 | 81 | 118 | 0.69 |
| 133 | M | 7y | N | N | ↓ | MHPA | E | NO | 2348 | 258 | 0.02 | 148 | 53 | 87 | 155 | 69 | 1.26 | 39 | 5.27 | 27.3 | 99 | 68 | 682 | 75 | 104 | 0.72 |
| 134 | M | 8y 3m | N | ↑↑ | ↑↑ | MHPA | E | NO | 3322 | 300 | - | 159 | 50 | 87 | 160 | 66 | 1.32 | 134↑ | 8.53 | 36 | 118 | 67 | 585 | 124 | 99 | 1.25 |
| 135 | F | 9y 3m | N | N | N | MHPA | E | NO | 2341 | 310 | 0.04 | 162 | 59 | 81 | 167 | 59 | 1.37 | 110↑ | 8.67 | 38 | 100 | 62 | 513 | 89 | 84 | 1.06 |
| 136 | M | 11y | N | N | N | MHPA | E | NO | 1085 | 324 | 0.03 | 164 | 66 | 83 | 177 | 54 | 1.54 | 127 | 5.74 | 39 | 117 | 68 | 558 | 67 | 86 | 0.78 |
| 137 | F | 12y | N | N | ↓ | MHPA | E | NO | 1922 | 288 | - | 157 | 41 | 101 | - | - | - | 125 | 3.9↓ | 34 | 110 | 57 | 664 | 52↓ | 76 | 0.68 |
| 138 | F | 21y 2m | N | N | - | MHPA | E | NO | 2793 | 288 | - | 179 | 36↓ | 121 | 130 | 103 | 1.17 | 108 | 14.1 | - | 132↑ | 85↑ | 297 | 67 | 97 | 0.69 |
| 139 | M | 23y 3m | ↑ | ↑ | - | MHPA | E | NO | 3260 | 294 | - | 161 | 48 | 103 | 155 | 87 | 1.18 | 56 | 8.2 | 39 | 94 | 64 | 270 | 109 | 70 | 1.56 |
| 140 | F | 23y 1m | N | N | - | MHPA | E | NO | 2407 | 294 | - | 182 | 73 | 82 | 222 | 58 | 1.41 | 138↑ | 7.76 | 38 | 130 | 75 | 286 | 95 | 166↑ | 0.57 |
| 141 | M | 25y 7m | ↑ | ↑ | - | MHPA | E | NO | 1685 | 300 | - | 160 | 60 | 82 | 183 | 97 | 0.85 | 90 | 4.5↓ | 32 | 130 | 82 | 263 | 115 | 53↓ | 2.17 |

Additional file: Characteristics of Hyperphenylalaninaemia patients with altered values of the biochemical parameters studied

P: patient; M: male; F:female; BMI: body mass index; WC: waist circumference; AC: arm circumference; BP: blood pressure; E:early; L:late;N: normal; Phe tol.: Phenylalanine tolerance; Phe med: annual median blood phenylalanine ;↑overweight. ↑↑ obesity. CPKU: classic phenylketonuria; MPKU: mild-moderate PKU; MHPA: moderate hyperphenylalaninaemia; Treat.: Treatment; E: Early; L:late; Total Chol.: total cholesterol; Trig: triglycerides; tHcy: total homocysteine; Met: Methionine
